# Using Fly Ash Wastes for the Development of New Building Materials with Improved Compressive Strength

**DOI:** 10.3390/ma15020644

**Published:** 2022-01-15

**Authors:** Maria Harja, Carmen Teodosiu, Dorina Nicolina Isopescu, Osman Gencel, Doina Lutic, Gabriela Ciobanu, Igor Cretescu

**Affiliations:** 1 Department of Chemical Engineering, Faculty of Chemical Engineering and Environmental Protection, Gheorghe Asachi Technical University of Iasi, 73 D. Mangeron Blvd., 700050 Iasi, Romania; gciobanu@tuiasi.ro; 2 Department Environmental Engineering and Management, Faculty Chemical Engineering and Environmental Protection, Gheorghe Asachi Technical University of Iasi, 73 D. Mangeron Street, 700050 Iasi, Romania; 3 Department of Civil and Industrial Engineering, Faculty of Civil Engineering and Building Services, Gheorghe Asachi Technical University of Iasi, 65 D. Mangeron Street, 700050 Iasi, Romania; dorina-nicolina.isopescu@academic.tuiasi.ro; 4 Civil Engineering Department, Faculty of Engineering, Bartin University, Bartin 74100, Turkey; osmangencel@gmail.com or; 5 Department of Material Chemistry, Faculty of Chemistry, Alexandru Ioan Cuza University, 11 Carol I Bd., 700506 Iasi, Romania; doilub@uaic.ro

**Keywords:** fly ash, alkali activated materials, properties, capitalization

## Abstract

Fly ash wastes (silica, aluminum and iron-rich materials) could be smartly valorized by their incorporation in concrete formulation, partly replacing the cement. The necessary binding properties can be accomplished by a simple procedure: an alkali activation process, involving partial hydrolysis, followed by gel formation and polycondensation. The correlations between the experimental fly ash processing conditions, particle characteristics (size and morphology) and the compressive strength values of the concrete prepared using this material were investigated by performing a parametric optimization study to deduce the optimal processing set of conditions. The alkali activation procedure included the variation of the NaOH solutions concentration (8–12 M), temperature values (25–65 °C) and the liquid/solid ratio (1–3). The activation led to important modifications of the crystallography of the samples (shown by powder XRD analysis), their morphologies (seen by SEM), particle size distribution and Blaine surface values. The values of the compressive strength of concrete prepared using fly ash derivatives were between 16.8–22.6 MPa. Thus, the processed fly ash qualifies as a proper potential building material, solving disposal-associated problems, as well as saving significant amounts of cement consumed in concrete formulation.

## 1. Introduction

The high rate of development in construction industries in the recent years resulted in the use of large amounts of cement [1] and the production is expected to remain around 4100 Mt/year or even increase in the next years [2]. The current technology in cement production involves large quantities of mineral resources and their processing at high temperatures, usually generated from burning fossil fuels. This fact places the cement industry in an undesired top of high carbon dioxide emission: for 1 t of cement, 0.58 t of CO_2_ is emitted [3]. Due to technologies enhancements, the CO_2_ emissions are expected to decrease to 0.48 t CO_2_ per ton of cement until 2030 [2]. A strategy in this respect is the use of alternative materials as cement feedstock substitutes. In the latest years, studies have been dedicated to finding substitute materials similar to cement binders [4,5], aiming to solve some economic and environmental problems by lowering the CO_2_ emissions by the decrease of energy consumption during the fabrication [6].

The alkali activated materials are new, low-cost binding materials prepared from metakaolin or from different solid wastes with similar compositions [7], which could act as excellent building materials with a notable potential use. The activation procedure consists of a treatment with alkali concentrated solution, which helps to solve some of the amorphous material and enrich the material in silica and aluminum oxide. 

The industrial development generates huge amounts of solid waste, damaging extended soil surfaces due to their disposal or accidental spreading and affecting thus the agriculture. The ground water contamination is also a potential threat, all of these acting harmful to the human health. The proper valorization of the industrial waste (by-products from the production of numerous commodities) reduces the discarding/neutralization costs, limits the environmental pollution and contributes to the industrial development on the basis of sustainability principles [8]. 

The alkali-activation of aluminosilicates delivers new binders with similar properties as cement. The sources of aluminosilicates can be several natural resources or industrial by-products, such as different ashes (from power plants, rice husk), slag and red mud [9,10]. The literature reports obtaining of alkali-activated binders from calcium and silicon-rich material, generating by activation calcium-silicate-hydrate (C-S-H); aluminum and silicon-rich material, transformed later in alumino-silicate hydrates and of calcium, aluminum and silicon-rich material, prepared as a hybrid alkaline binder [11,12]. Fly ash, with its convenient valorization and environmental friendliness, has a wide addressability in both research and economic communities. A simple search on Google Academic by using “cheap cement replacement materials alkali” gives 17,100 results published in the last 5 years (from 2017).

Substantial amounts of fly ash generated in the energy production from solid fuels have major environmental and health impacts. This snag could be avoided by the fly ash revaluation. Fly ashes could work as precursors for new building materials, this process being a smart route for their convenient recycling, in the meantime with the production of new materials with good durability and high mechanical strength [13,14]. Fly ashes are a major industrial waste, which, due to its chemical composition and hydraulic properties, can be a source of new raw materials useful in various fields, such as concrete fillers, geopolymers, cement substitutes, catalysts, adsorbents, land stabilizers, ceramics, heavy metal removal from wastewaters and zeolites [15,16,17]. The chemical and physical properties of fly ash vary according to the coal source and burning conditions. The valorization field depends on the ash properties [15,18], therefore, deeper research for new capitalization strategies areas is mandatory. In this context, the present study is focused on understanding the influence of particle size on the mechanical properties of the concrete prepared using also alkali activated fly ash in the mixture. The results could be extended for the ashes formed from coals based on lignin coming from other geographic areas. 

Fly ashes are mixtures of silica, alumina and iron oxides, having three-dimensional alumino-silicate networks. Their alkali-activation processes are often used for their capitalization [14], due to their behavior being quite similar to the ordinary Portland cement. The success key for the development of materials with binding properties is the activation regime, comprising the precursors hydrolysis at temperatures up to 100 °C (direct activation) or over 500 °C (hydrothermal activation), followed by gel formation and polycondensation [16]. 

Puertas et al. [19] prepared alkali-activated materials by activating a fly ash/slag with sodium NaOH solutions (2–10 mol/L), at temperatures between 25–65 °C, for a time duration of 5 h. The results revealed that the NaOH concentration, the temperature and the fly ash/slag ratio influenced the compressive strength of the resulted material. Bakharev [20] established that at ambient temperature, a longer curing time is favorable for the alkali activated material; on the other hand, the elevated temperature shortens the curing time, in parallel with the increase of the strength. Chindaprasirt et al. [21] demonstrated that the curing of fly ash class C for one hour did not exert a negative effect over the mechanical strength. 

Ibrahim et al. [22] reported that the chemical composition of the raw materials, the alkaline solution concentrations and the curing conditions influenced the hardening properties of alkali-activated binders. By using natural pozzolan and NaOH (8–14 mol/L), a dense microstructure with maximum mechanical strength was obtained. 

Bocullo et al. [23] demonstrated that the highest compressive strength for alkali activated of low calcium fly ash was reached during a treatment leading to a material with a SiO_2_/Na_2_O ratio between 1.5–2.3. 

Nguyen et al. [24] recommended the alkali treatment of fly ash with sodium hydroxide and sodium silicate at ambient temperatures, for obtaining a higher compressive strength.

Most research in the last few years have been focused on studying the effects of the raw materials composition, alkali types and composition, temperatures, solid–liquid ratio, etc. on the mechanical strength of alkali-activated materials obtained from fly ash. 

One of the reasons we consider this research valuable is that it was performed on a fly ash obtained at the power station for the agglomeration of Iasi, Romania, which produces electrical and thermal energy mainly from coal (the approximate number of residents in the town and close environment is around 500,000 people). Therefore, large amounts of this waste are produced and its management over the last 30 years has been only open-air disposal. Our aims were to get rid of this potential pollutant (due mainly to the large amounts produced) in a green strategy, and to substitute some of the high energy-consuming cement from the preparation recipe of concrete, while preserving the resistance parameters requested. The previous investigations indicated that the liquid–fly ash ratio, the molar concentration of the alkaline solution and the temperature are the most important factors in determining the size and morphology of the treated materials [14]. In this study, sodium hydroxide was used for the alkaline activation of fly ash, based on a low-cost procedure, aiming to turn this waste to an alternative cement material; a working parameters optimization was performed in this respect. The particle size distribution of the materials was correlated with their compressive strength. According to the literature search performed, this is the first time when the nanoparticles characteristics prove to influence the mechanical properties of the binder similar to cement. 

## 2. Materials and Methods

### 2.1. Materials Synthesis

The fly ash for this study was collected in November-December 2020 from the electric filters of the main power plant situated in the proximity of Iasi, Romania. NaOH (Merck) was used as an activation species: solutions between 8–12 mol/L were prepared by using distilled water.

The experimental procedure consisted of mixing the fly ash with the alkali solutions and stirring for 4 h, at either 25 or 65 °C. The solid was subsequently filtered, washed and dried. The structural investigation of the initial solid revealed that it was a class F fly ash (Fe-rich clay), the same as we found in some previous studies [13,25]. The solid-to-liquid ratios optimization meant choosing the minimum necessary alkali solution for the thorough wetting of the ash and the proper feedstock transformation, with a minimum amount of solution. Solid/liquid ratios used were 1:1, 1:2 and 1:3.

A main series of alkali activated materials (AAM) were obtained in diverse operating conditions, by following the experimental design matrix presented in Table 1.

The Si and Al contents from the samples used for calculating the Si/Al ratios from the last column were determined by EDAX analysis. 

### 2.2. Methods

The chemical, morphological and mechanical properties of the initial fly and of the AAMs materials were investigated by Scanning Electron Microscopy–SEM (Quanta 3D–AL99/D8229–FEI Company, Hillsboro, OR, USA) and X-ray diffraction analysis–XRD (X’Pert PRO MRD X-ray diffractometer–PANalytical, Malvern, UK). The particles size distribution was determined by using a Shimadzu SALD 7001 laser diffraction analyzer (Shimadzu Scientific Instruments, Durham, NC, USA). The samples’ preparations for the analysis were made by dispersion in acetone, ensuring their good dispersion and allowing to obtain equivalent diameters values close to the ones found in a dry state (no swelling).

The technological parameters investigated were the Blaine surface (determined by measuring the air flowing resistance through a porous bed prepared in standard conditions, as stated in SR EN 196-1: 2016 [26]) and the determination of the compressive strength of the concrete prepared with alkali processed fly ashes. According to the concrete preparation standards [27,28], the mixture for obtaining the C16/20 concrete consists of 280 kg cement, 567 kg fine sand (0–4 mm i.d.), 252 kg intermediate sand (4–8 mm), 400 kg half-coarse sand (8–16 mm), 525 kg coarse sand (16–31.5 mm) and 161 L of water. On the dry base, the cement means only 13.8% from the mass. Thus the proportion used in our tests replaced about 75% of the cement with processes fly ash. The materials listed in Table 1 were used by preparing a mixture of sand, cement (3.8%) and 10% AAMs. The mechanical properties investigation was carried out by applying the standard procedure used in cement-containing materials, on a cube specimen, by using the universal testing machine. The compressive strength was performed according to SR EN 196-1: 2016. Five parallel measurements were performed for confirming the measurements reproducibility. 

## 3. Results and Discussions

A comprehensive characterization of the AAM materials used in this study by energy dispersive X-ray spectroscopy (EDAX), Fourier transform spectroscopy (FTIR) and scanning electron microscopy (SEM) analysis had been already published in a previous work [14]. Therefore, the main purpose of the current paper is to correlate the mechanical properties of the concrete (prepared using the samples listed in Table 1), with the particle size distribution.

### 3.1. Material Characterization

The chemical composition of the fly ash was performed by EDAX and revealed that the material consisted of Si, Al, Na, Fe and Ca [29]. 

The alkali treatments applied brought important changes in the chemical composition of the samples; in the last column of Table 1, the values indicate that the Si/Al ratios strongly depend on the treatment conditions. Since aluminum is relatively easily transformed in aluminate in high alkaline conditions, the dealumination of the initial fly ash (the increase of the Si/Al ratio) could explain the mechanical properties improvement of the new synthesized materials. 

**Figure 1:**
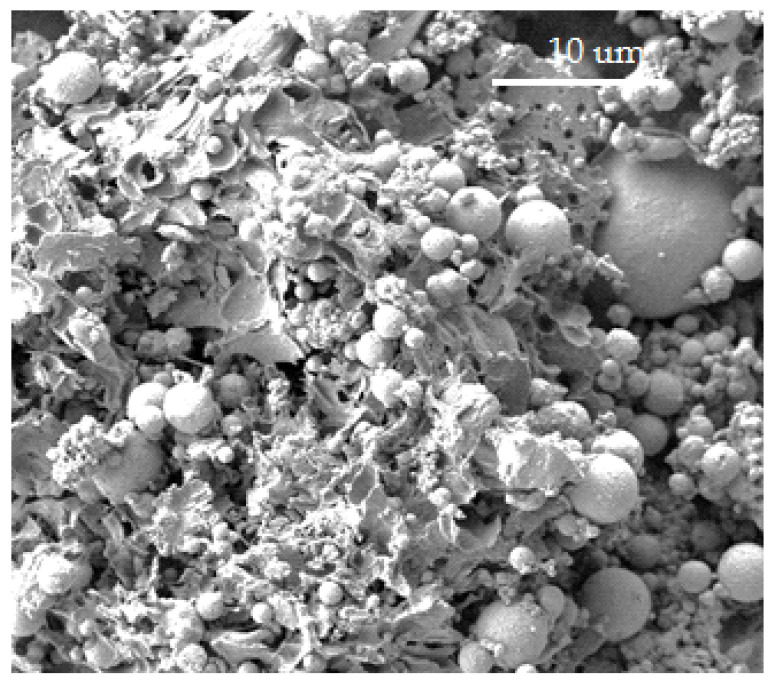
SEM image of the genuine fly ash.

The SEM image of the fly ash from the thermal power plant of Iasi Municipality (Figure 1) indicate the presence of numerous spherical particles, with different diameters comprised between 1–10 μm, embedded in a pseudo-matrix of highly irregular, strongly broken cages with thin walls [29,30]. 

**Figure 2:**
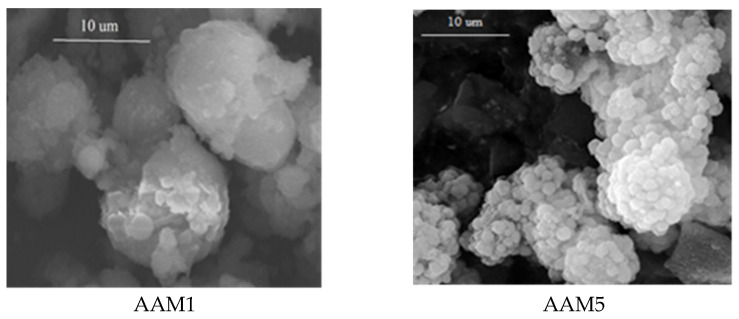

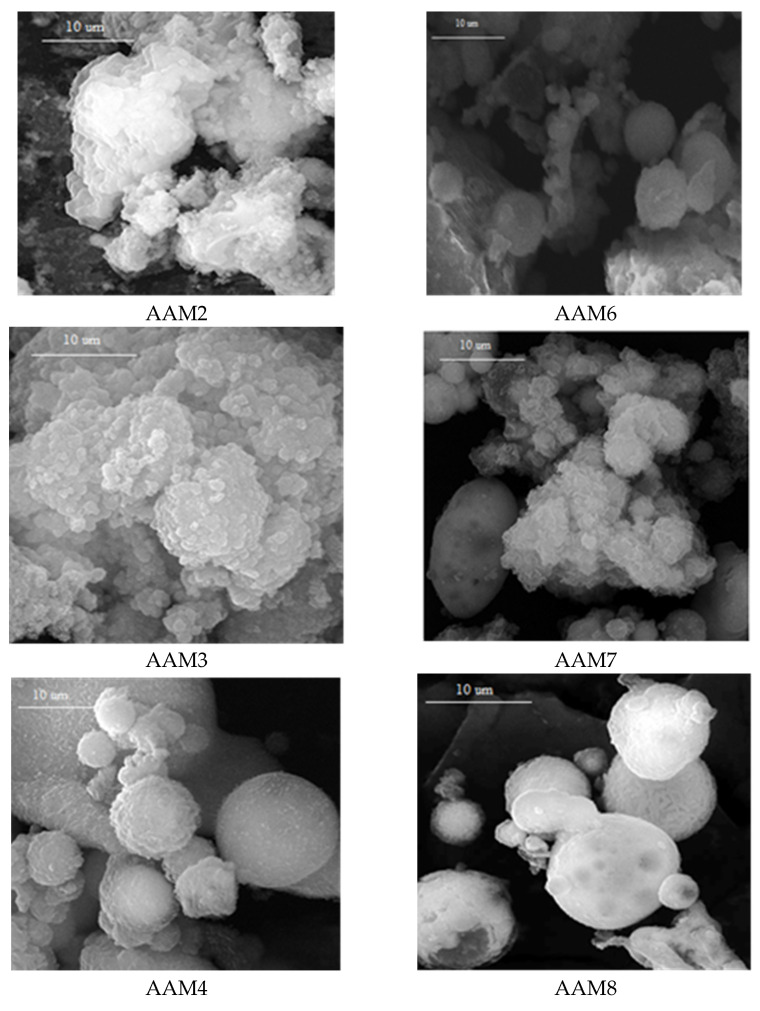
SEM micrograph of the fly ash samples processed by alkali treatments.

According to the SEM images of the AAMs samples (Figure 2), the morphology of the processed ash samples is changing dramatically in terms of particles shapes, sizes and surface roughness. The initial irregular matrix disappears almost totally, being dissolved by the alkali solution and/or delivering the feedstock for the subsequent recrystallization reactions. Some of the initial spheres keep almost unchanged the outer surface aspect, while in other cases, a strong increase of the deepness and number of asperities is noticed. As a general trend, the higher treatment temperatures seem to dissolve partially also the initial spheres from the ash, either by making their surface rougher or even develop flake-like smaller particles almost detached from the surface. In all the images, the smooth, spherical particles from the initial ash became quite rough during the treatment and indicate that they consist of smaller particles with sheet-like and polyhedral shapes tightly associated. 

**Figure 3:**
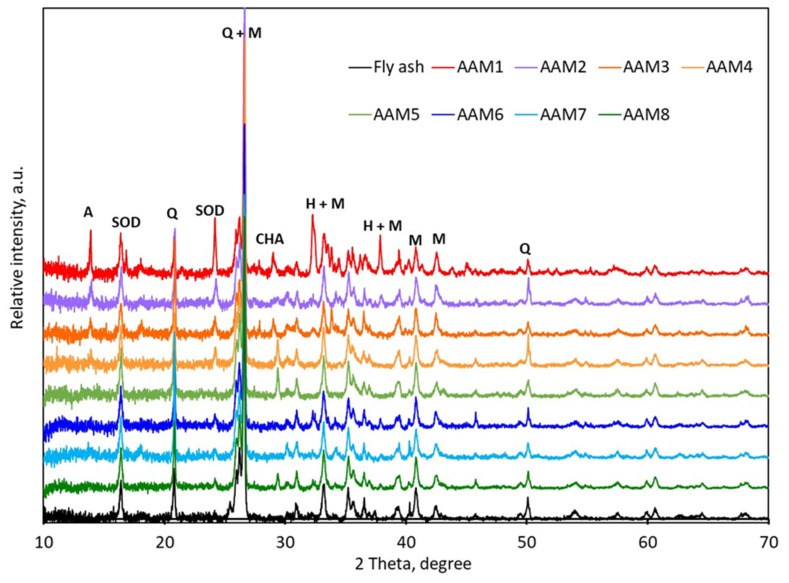
XRD patterns for fly ash and selected processed materials (Q—quartz, M—mullite, H—hematite, A—linde type A zeolite, CHA—chabazite, SOD—sodalite).

These dramatic observed changes observed by the microscopic analysis required a careful and detailed analysis of the crystalline phases from the alkali-treated solids. The XRD patterns of the series of samples are presented in Figure 3. The initial fly ash contained crystalline quartz (Q), identified by the (101), (110) and (112) plans giving maxima at 2 theta values of 20.9; 26.6 and 50.1°, respectively (SiO_2_, JCPDS 05–0492); mullite phase (3Al_2_O_3_2SiO_2_), assigned by the maxima at 26, 26.2, 33.2, 35.2, 40.8, 42.6 (230) and 60.6°, corresponding to the (120), (210), (220), (111), (121) (331) plans [31], (JCPDS 15–0776) and to hematite (H) (Fe_3_O_4_, JCPDS 19–0629), identified by the (104), (110) and (116) plans, giving signals ay 33, 35 and 54°, respectively. 

The alkali treatments applied to obtain the AAM series of materials also have very important effects in terms of crystalline phases formation. Small amounts of new aluminum silicate crystalline phases of zeolite type were highlighted in the processed materials, respectively: sodalite (SOD), identified by the (211) and (310) plans (24.3 and 31°); chabazite (CHA), identified by the (3-1-1) and (310) plans (30 and 30.2°) and sodalite (211) and (310) plans (24.2 and 32°) [32]. However, the initial quartz, mullite and hematite remained the majority crystallographic phases from the products. These findings are in line with the literature [17,23,33].

**Figure 4:**
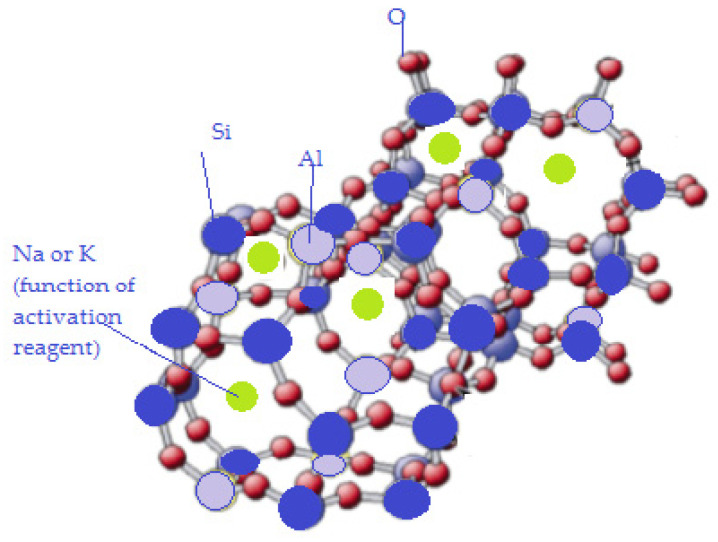
Zeolite precursor formed in the alkali activated process.

The strong alkali medium and especially the higher temperature values are favorable to zeolite formation; their framework consists of tridimensional [SiO_4_] and [AlO_4_] alternating units, generating micropores (molecular size range ordered voids), in which the aluminum-containing units could have only silicon-based units as first neighbors (meaning that the Si/Al ratio is always over 1). Further, the higher temperature of the alkali treatment was favorable for the zeolite formation. The tetrahedric aluminum units from the network bear a network anionic charge, therefore, compensation cations are necessary. In most cases, sodium and potassium ions are found as compensating cations. Up to a certain level, they also act as zeolitic structure templates, due to the fact that they are surrounded by a high number of water molecules. A sketch of the incipient generation of zeolite framework is given in Figure 4.

Due to the small size of the granules enclosed, Portland cement is a material with high surface area. This property defines the water content for reaching a normal consistency of the concrete, as well as in defining the mechanical properties of the resulted material. Previous research indicates that the particle diameters in cements can vary between 0.05–5 µm [14]. The SEM images of the fly ash in Figure 1 suggest that the particle size of the processed ashes renders them proper for the inclusion in the concrete. The specific surface areas determined by the Blaine method for Portland cement, fly ash and processed fly ash (Samples AAM1–AAM8) are presented in Table 2.

The Blaine surface value of the initial fly ash was less than a half compared to that of the cement, therefore its use directly in the concrete formula is not easy to presume. The values of the Blaine surface vary between 326–499 m^2^/kg, strongly depending on the alkali treatment conditions. The processed samples, excepting AAM4 and AAM7, have Blaine surface areas in the range of the Portland cement.

### 3.2. Mechanical Characterization of the Synthesized Samples

In the literature, there are mentions that the compressive strength values of the material are affected by the fly ash modification conditions [14]. The variation of the compressive strength values is explained especially on the basis of the Si/Al ratio [34]. On the other hand, there is also a relationship between the polycondensation products from the reactions during the mortar formation and the value of the compressive strength. The compressive strength values of the materials containg AAMs (tested according to SR EN 196-1:2016 [26]) are shown in section Modeling and Optimization Process.

A review concerning the influence of the preparation conditions published by Ng et al. [35] indicated that, according to Pavithra et al. [36] the low amounts of water in the preparations were favorable relating to the compressive strength of the obtained concrete. Our experimental results mainly indicate that the liquid-to-solid ratio for the ash processing exerts an important influence on the values of the compressive strength; but, in our case, the samples prepared at a liquid/solid ratio of 3:1 (excepting AAM1 versus AAM2), led to materials with better behaviors than the corresponding ones prepared at a ratio of 1:1. These findings seem to be in opposition with the fore-cited works. However, Pavithra et al. [36] have performed just an alkali treatment before including the fly ash in the concrete recipe (also optimized within their study), i.e., all the alkali was finally included in the concrete formula. In our study, the alkali solution was removed before using the processed fly ash in concrete preparation formula. In the meantime, the deepness of the transformations performed by us were larger, since samples AAM1–AAM4 were prepared at 65 °C. In these harsher conditions, the geopolymerization reactions were much favored.

The opposite trend between these series of samples indicates that the treatment temperature effect is extremely important. A liquid/solid ratio of 3, the value of NaOH solution concentration of 12 and the treatment temperature of 65 °C are the most severe conditions for the ash fly treatment, which is in line with the results in reference [37]. Since the compressive strength decreases after this set of conditions, it suggests that an optimum could be defined after a modelling of the processing.

**Figure 5:**
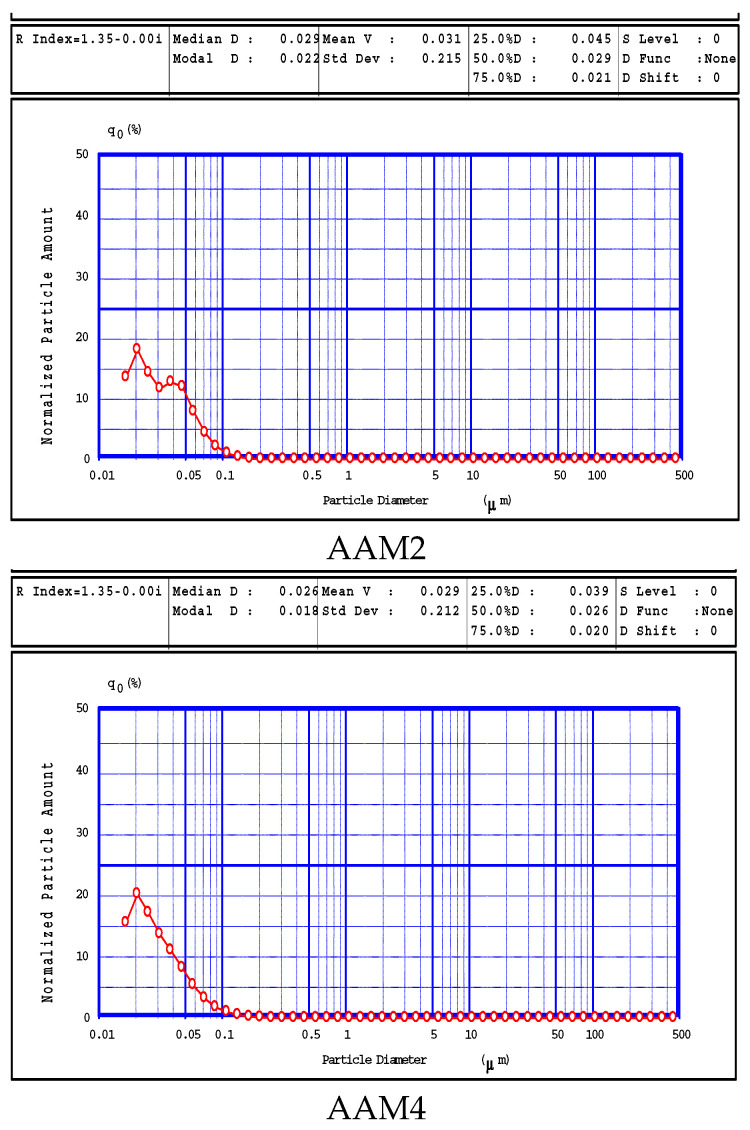

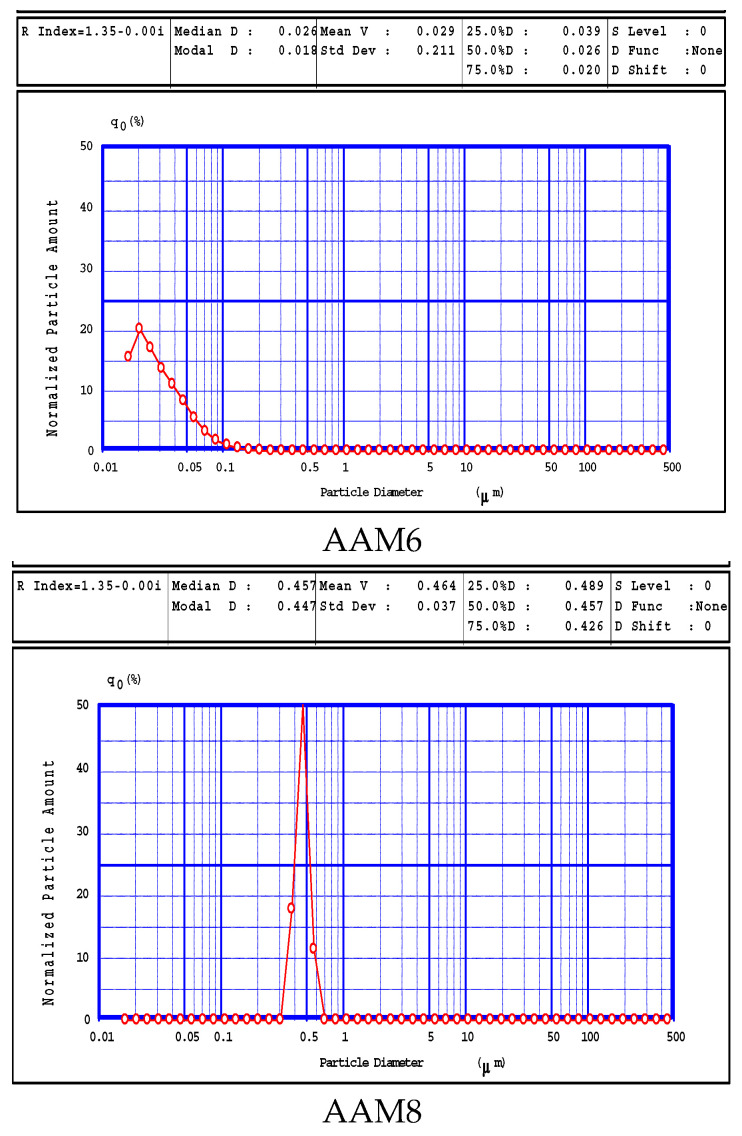
Particle size distribution of several samples.

Since the materials prepared at a liquid to solid ratio of 1:1 had the most interesting behaviors (maximum and minimum values of the compressive strength values from the experimental series), we have performed the measurement of the particle size distribution for the samples AAM2, AAM4, AAM6 and AAM8. The results are presented in Figure 5.

The particle size distribution for the samples AAM2, AAM4 and AAM6 are very similar to each other. The elementary particles detectable have diameters below 0.1 μm and the distribution curve shape is asymmetric and wide. A characteristic median diameter of 0.026 μm and modal diameter of 0.018 μm can be defined for AAM4 and AAM 6 samples. The particle size distribution for sample AAM2 has a somehow similar shape, but the curve is rather irregular; the median diameter value deduced by the data processing of 0.029 μm does not define a characteristic value as for samples AAM4 and AAM6. The results for sample AAM8 are dramatically different: the particle size distribution is described by a very narrow curve showing a median diameter of 0.457 μm and the modal diameter of 0.447 μm. The particle size distribution confirms the results obtained by SEM analysis, indicating that the smooth spheres seen by microscopy are really individual particles with very similar radius values. These particles result during the treatment in the presence of low volume of NaOH solution at 25 °C only are formed just by the dissolution of the pseudo-matrix containing the spherical particles from the initial fly ash. The low temperature value and the diluted NaOH (8M) could not dissolve parts of the spheres included in the fly ash, as in the other samples. These deeper reactions required either a more energetic set of conditions (temperature of 65 °C, AAM2 and AAM4) or a 3:1 liquid/solid ratio.

The narrow particle size distribution encountered for sample AAM8, as shown by the results in Figure 5, indicate that the particles are bigger and have a very narrow particle distribution, as shown by the very close values of the median, modal and mean diameters values.

The above results indicate that important differences occur between the size distribution and uniformity of the particles from the fly ash after alkali treatments operated in different conditions of temperature, NaOH concentrations and liquid/solid ratios. The production of solids consisting in particles of tens of nanometers (highlighted by the particle size distribution curves) associated in agglomerations with sizes up to 10 μm (seen on SEM images) led to their good valorization as substitutes of cement in the concrete, by their potential to generate in specific mixtures materials with compressive strength values of over 20 MPa.

### 3.3. Modeling and Optimization Process

**Figure 6:**
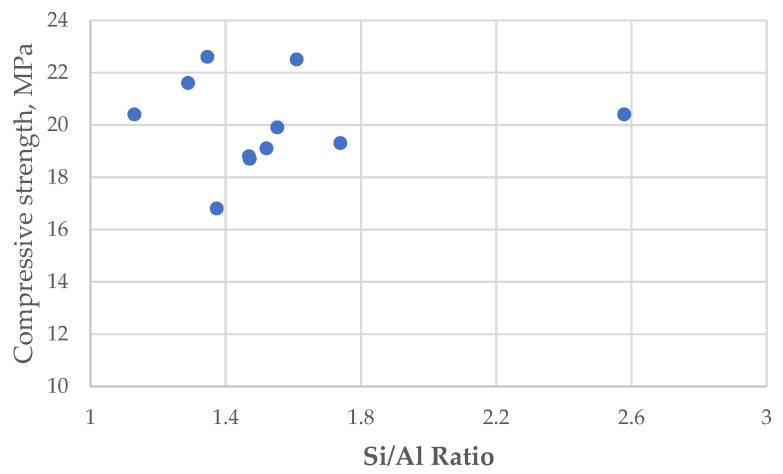
The influence of Si/Al rate in different experimental conditions on the the compressive strength of synthesized materials.

The mathematical modeling of the compressive strength values depends on the fly ash activation parameters, from which the most significant were selected taking into consideration the complex influence of Si/Al ratio toward the compressive strength of the prepared materials (see Figure 6).

The values from Figure 6 suggest that there is no direct correlation between the value of the compressive strength and the Si/Al ratio from the processed fly ash, but clearly show that high dealumination (high Si/Al ratio) is not favorable. This fact was another reason to perform a parametric optimization study.

Even if there are several parameters which could influence the synthesized material properties, only the following parameters were selected based on the previous preliminary tests: liquid/solid ratio–X1, NaOH concentration–X2 and temperature value–X3, which were tested at 3 levels (central (0), inferior (−1) and superior (+1)) as are centralized in Table 3.

The experimental design [38,39], coefficient determination of the obtained models, data analysis and 2/3D graphical plots were obtained using NemrodW software (Version 2000-D, LPRAI, Marseille, France). The experimental design matrix, according to a full three-level Box–Behnken factorial design, was obtained and the system response (Y_1_), in term of compressive strength was experimentally determined. The system response was fitted by an empirical second-order polynomial model, but unfortunately the established regression coefficients for quadratic and interaction terms, were not validated from the statistical point of view. Therefore, a linear empirical polynomial model was verified based on the experiments necessary for 2^3^ factorial experimental matrix design, where the factors were modified at two levels (superior and inferior), using the previous data only to check the experimental reproducibility at the central level. This new matrix design is presented in Table 4.

According to the modeling methodology for the first-order model, based on the above factorial matrix design with 3 parameters (X1, X2 and X3) and their interaction terms, the mathematical expression can be written as a polynomial equation:

Y1 = b0 + b1X1 + b2X2 + b3X3 + b12X1X2 + b13X1X3 + b23X2X3 + b123X1X2X3(1)

where: b0 is the average value of the result; b1, b2 and b3 are the linear coefficients; and b12, b13, b23 and b123 represent the interactions coefficients. The variables X1, X2 and X3 represent the parameters considered in the developed model. Combinations of these (such as X1X2) represent interactions between the individual parameters.

The complex response function obtained from the presented data in Table 4 is given by the Equation (2):

Y1 = 5.98125 − 0.38125X1 + 0.864062X2 + 0.0787500X3 + 0.254688X1X2 + 0.0812500X1X3 + 0.0034375X2X3 − 0.0121875X1X2X3(2)

The significance of the model coefficients and the model validity was successfully confirmed by the statistical tests, for a Probability <0.05, using the above-mentioned software. A good correlation between the predicted values and the measured values for the mechanical properties in terms of the compressive strength was obtained for this linear model.

Using the developed model for the compressive strength (Fc) and the experimental operational conditions, the optimum of these could be localized on the surface response and could estimate the main effects for each parameter, as well the interaction effects of them. Based on the model equation, X2 and X3 have a positive effect on the response, due to the positive values of the coefficients b2 and b3, while X1 has a negative effect. The mechanical properties of the prepared materials increase when the above-mentioned parameters (X2 and X3) changes from low level to high level, and falls when parameter X1 passed from the low levels to the high levels. It should be noted that all linear coefficients have a subunit value, which means the determining effects are not very strong (even if they are positive or negative), but from all of them the most significant seems to be the concentration of NaOH (X2). All interaction effects of the three parameters (in the low level and the high level) have a positive effect (in the following order: X1X2 > X1X3 > X2X3), with the exception of combined interactions of X1X2X3, which has a small negative effect (almost neglecting).

**Figure 7:**
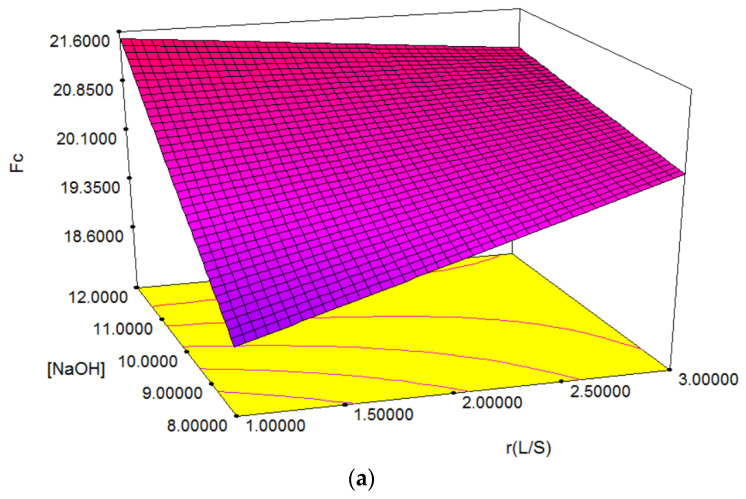

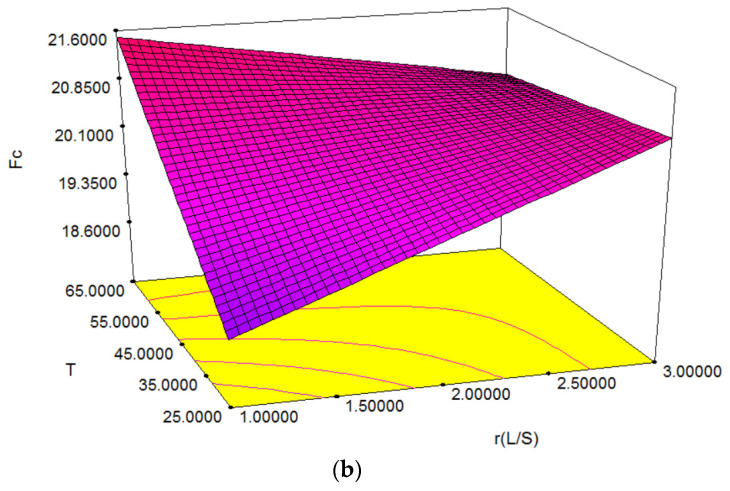
Response surface Fc as a function of parameters Xi and Xj (for Xk = constant): (**a**) Fc = f(X1, X2); (**b**) Fc = f(X1, X3).

In order to provide a better explanation of the effects of the independent variables and their interactions, (2D) and (3D) response surface plots were drawn as a function of three parameters at a time, holding the third parameter as fixed. The Graphical representations (Figure 7a,b) show the Fc behavior under the simultaneous change of the three variables, while fixing the third one.

The 2D and 3D surface plots show that the maximum value for Fc was obtained under alkaline media conditions (around pH ~12), for a S/L ratio around 1, and a temperature of 65 °C. The optimal experimental conditions were obtained by analyzing the response surface contour plots (graphical analysis).

### 3.4. Perspectives

The fly ash capitalization is widely approached in literature: this waste could act as a valuable precursor for cement containing materials due to its low cost, large availability, and excellent reduction of the evil environmental impact when its uncontrolled spreading is avoided [40,41]. The sources and varieties of coals used as fuels, the specific burning conditions, the collection technical solutions and the disposals temporary conditions influence the elemental composition and the crystallographic characteristics of the ashes. The conditions of alkaline attack influenced the nature of the obtained products. The long attack time determines the dissolution of aluminum oxide and the formation of silica-rich products, favorable over the final resistance properties of the materials. On the other hand, the alkaline activator has a significant influence [42], and the polymerization degree depends on the SiO_2_:Na_2_O ratio [43]. The most commonly ash activators are sodium hydroxide, potassium hydroxide and sodium silicates. The temperature has an important influence on the reaction kinetics and on the final products properties.

Each initial material must be thoroughly investigated for finding the optimal conditions of alkaline attack and obtaining modified products with proper properties according the foreseen practical applications.

This kind of research extension towards other industrial byproducts and finding alternative activators that can be advantageous in terms of ecological impact and/or final price. The need of extra-silicon in the material formulation could be filled by using ultrafine particles (nano-silica) to reach the optimal SiO_2_:Na_2_O ratio. The British Standards, for instance, allow the commercialization of low carbon cements obtained by the alkali activation of waste and encourage their use as construction materials. The environment problems could be encountered if more countries would update their legislation in accordance to corresponding specifications [44].

## 4. Conclusions

The experimental results show that fly ash could be used as a building material substitute after a simple processing. Fly ash can be valorized after an alkali treatment, thus reducing the environmental pollution.

The objective of this study was to establish the influence of the operating conditions of the alkali action, on the formation of the most favorable nano-sized particles for obtaining binders substitutes for the fabrication of concrete with high compressive strength. The alkali activated materials are an efficient, low-cost alternative to Portland cement, with important benefits regarding the consumed energy, directly connected to the carbon dioxide emission.

The optimization of the preparation conditions by applying a factorial design allowed us to obtain the optimum by performing the reduced number of experiments. The experimental data indicated that the optimal activation occurs at high NaOH solution concentrations (around 12 mol/L), high temperature value (65 °C) and L/S ratios around 3. The optimal processing conditions of fly ash led to the formation of nanoparticles of less than 0.1 μm, highly agglomerated in pseudo-spherical aggregates with median diameters between 20–30 nm. Their inclusion in the preparation of concrete as a cement replacement led to materials with compressive strength values of over 20 MPa.

The proposed conditions allow the reduction of the abundant industrial waste from a thermal power plant, available in considerable amounts.

## Figures and Tables

**Table 1:** The materials selected for investigation.

Sample	L/S Ratio	C_NaOH_, M	Temperature, °C	Si/Al
AAM1	3:1	12	65	1.553
AAM2	1:1	12	65	1.346
AAM3	3:1	8	65	1.289
AAM4	1:1	8	65	2.579
AAM5	3:1	12	25	1.610
AAM6	1:1	12	25	1.130
AAM7	3:1	8	25	1.739
AAM8	1:1	8	25	1.374

**Table 2:** The Blaine specific surface area for synthesized materials.

Sample	S_Blaine_, m^2^/kg
Portland Cement	472–560
Fly ash	209.2
AAM1	499.0
AAM2	478.3
AAM3	479.0
AAM4	326.2
AAM5	477.0
AAM6	465.1
AAM7	256.4
AAM8	473.2

**Table 3:** The range and levels of experimental variables (parameters).

Coded Variables	Parameters	Coded Level		
		−1	0	+1
X_1_	Liquid/Solid ratio	1	2	3
X_2_	NaOH concentration	8	10	12
X_3_	temperature (°C)	25	45	65

**Table 4:** The experimental matrix design based on 2^3^ factorial methodology.

Run No.	Sample No.	X1 L/S Ratio	X2 C_NaOH_ [M]	X3 Temperature [°C]	Si/Al Ratio	Y1 Fc [MPa]
1	AAM1	3:1	12	65	1.553	19.9
2	AAM2	1:1	12	65	1.346	22.6
3	AAM3	3:1	8	65	1.289	21.6
4	AAM4	1:1	8	65	2.579	20.4
5	AAM5	3:1	12	25	1.610	22.5
6	AAM6	1:1	12	25	1.130	20.4
7	AAM7	3:1	8	25	1.739	19.3
8	AAM8	1:1	8	25	1.374	16.8
9	AAM9	2:1	10	45	1.471	18.7
10	AAM10	2:1	10	45	1.469	18.8
11	AAM11	2:1	10	45	1.521	19.1

## Data Availability

The data presented in this study are available on request from the corresponding author.
